# Editorial: Why Have Cortical Layers? What Is the Function of Layering? Do Neurons in Cortex Integrate Information Across Different Layers?

**DOI:** 10.3389/fnana.2018.00078

**Published:** 2018-10-09

**Authors:** Kathleen S. Rockland, Javier DeFelipe

**Affiliations:** ^1^Anatomy and Neurobiology, School of Medicine, Boston University, Boston, MA, United States; ^2^Universidad Politécnica de Madrid, Madrid, Spain; ^3^Instituto Cajal, Madrid, Spain

**Keywords:** isocortex, insular cortex, olfactory, periallocortex, cell types, receptors, reeler, GABAergic connectivity

This research topic brings together 14 articles dealing with the laminar organization of the neocortex. By convention, there are six cortical layers, but this organization may vary throughout the cerebral cortex of a given species or between species: many regions lack one or more layers, whereas in other regions there is good reason to consider more than six layers. An important perspective on neocortical lamination is thus the recognition that the cortical mantle is not homogeneous. With this in mind, we open with three articles discussing evolutionary comparisons of neocortex with non-neocortical, three-layer areas; namely, the hippocampal formation (Mercer and Thomson), periallocortical areas (Insausti et al.), and olfactory cortex (Shepherd and Rowe). Mercer and Thompson summarize and compare the development of the neocortex and hippocampus (mostly of the cat and monkey), the characteristics of their neurons, the circuits they form and the ordered, unidirectional flow of information from one hippocampal region, or one neocortical layer, to another. Insausti et al. focus on the main distinctions among the allocortex, periallocortex and isocortex, based on anatomical differences, in particular the number of layers, overall organization, appearance and connectivity. Finally, Shepherd and Rowe, discuss the possible existence of a basic cortical circuit that had its origin in the three-layer forebrain cortex of the ancestral amniote, that was conserved in non-avian reptiles, and that became elaborated in mammalian six-layer neocortex.

The laminar location of cortical neurons—their cell bodies—is determined during development. Two articles treat the developmental context. Popovitchenko and Rasin discuss transcriptional and post-transcriptional regulatory mechanisms related to pyramidal neuron genesis and migration in mice. González-Arnay et al. present new data on the derivation and migratory pathways of principal neurons in the insula cortex of fetal human brains, between 9 and 25 gestational weeks, including how these factors can relate to cytoarchitectonic diversity in the adult.

Cortical layers are deceptive—there and not there (as, the Cheshire Cat); conspicuously identifiable, but only when one stays within the classical cytoarchitectonic criteria of soma size and neuronal packing density. Notably, as reviewed by Narayanan et al. multiple cell types intermingle within the layers, and dendrites and local axons extend across multiple layers. These authors identify several cross-laminar patterns and propose 10 excitatory cell-type patterns with an orderly relation to laminar landmarks in rat barrel cortex. Larkum et al., reviewing fMRI data, similarly make a case that a simplified schema of “point neurons” is inadequate to convey the distributed functional properties of the neuronal arborization as this samples across layers.

Taking a global perspective, Opris et al. present data from biomorphic multielectrode arrays in support of laminar specific specializations for perceptual or executive circuits in the dorsolateral prefrontal cortex of nonhuman primates, and further argue on this basis for inter-laminar integration.

What about cortical connections? These are well-known to evince a laminar-specific bias, although important details, variability, and functional significance remain only sketchily understood. In their overview based on mouse visual cortex, D'Sousa and Burkhalter talk about layer-specific patterns of extrinsic connections, but suggest these might observe a gradient-like organization, rather than a sharp distinction of “feedforward” (“driving”) and “feedback” properties (respectively, “driving” and “modulatory”).

In the interaction of pre-synaptic inputs and their postsynaptic targets, receptors play an important role, and their orderly distribution is suggestive of layer-specific (or layer-biased) signaling mechanisms. These are discussed by Santana and Artigas (review: rat medial prefrontal cortex) and Zilles and Palomero-Gallagher (original research: human). These latter discuss the interesting result that receptor densities are not uniform across areas but segregate into definable area-specific clusters (a.k.a.,“fingerprints”). Radnikow and Feldmeyer further review layer- and cell-type specific differences in the effects of neuromodulator receptors on excitatory neurons of different cortical layers, with considerations of axonal projection patterns and their target structures.

Fish et al. use new quantitative fluorescent microscopy techniques to address the amount and type of GABAergic inhibition in different layers of human postmortem tissue of prefrontal cortex. Findings are discussed in the context of the laminar distribution of differentiable GAD+ terminations, GABAergic cell bodies, and their intracortical local connectivity.

Finally, in a paradoxical conclusion, Guy and Staiger review the relatively normal functioning of reeler mice, despite the severe disruption of their cortical lamination. They conclude that cortical layers *per se* are not an essential component for *basic* perception and cognition.

In summary, different cortical layers have distinct transcriptomic profiles, neurochemical attributes, connectivity patterns, number and types of synapses and many other structural attributes. There is evidence for cross-laminar integration. Nevertheless, results based on anatomy, or physiology or imaging leave largely unanswered, What is the function of each cortical layer? What do the different layers do? Thus, while lamination can be seen as inherent to neocortical organization, important questions remain open or only partially answered. Moving forward, can we refine these questions to be more approachable?

## Author contributions

JD and KR collaborated together in initiating this Topic, and in writing the Editorial and Introduction.

### Conflict of interest statement

The authors declare that the research was conducted in the absence of any commercial or financial relationships that could be construed as a potential conflict of interest.

